# Supplementary figures and images for: SARS-CoV-2 protein structure and sequence mutations: Evolutionary analysis and effects on virus variants
Source: PLoS One. 2023 Jul 20;18(7):e0283400. doi: 10.1371/journal.pone.0283400 (PMC10358949; doi:10.1371/journal.pone.0283400)

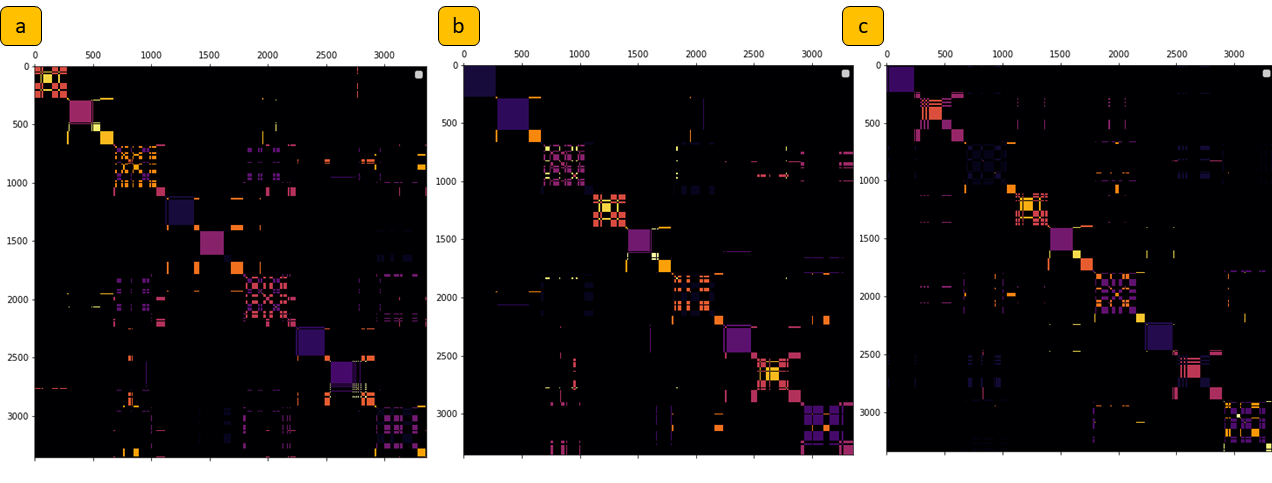

Supplement: S1 Fig — Clustermap plot for a) Wild Type, b) Delta, and c) Omicron1 variant. In a clustermap plot, communities are mapped in a matrix and visualized by means of heatmap. (TIF) [file pone.0283400.s001.tif]

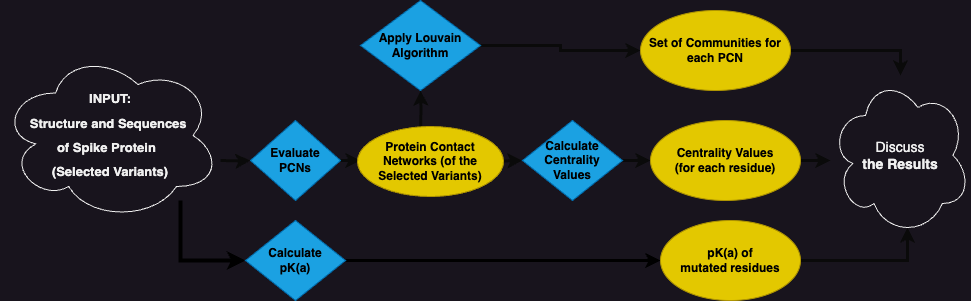

Supplement: S1 Graphical abstract — (PNG) [file pone.0283400.s003.png]
