# Supplementary material for: SARS-CoV-2 protein structure and sequence mutations: Evolutionary analysis and effects on virus variants
Source: PLoS One. 2023 Jul 20;18(7):e0283400. doi: 10.1371/journal.pone.0283400 (PMC10358949; doi:10.1371/journal.pone.0283400)
Supplement: S1 File — (DOCX) [file pone.0283400.s002.docx]

Supporting Information

More information can be found at the following link https://github.com/UgoLomoio/SARSCoV2_variants_PCN which contains code, high-quality figures and data used for this work.
